# Schizotypal traits across the amyotrophic lateral sclerosis–frontotemporal dementia spectrum: pathomechanistic insights

**DOI:** 10.1007/s00415-022-11049-3

**Published:** 2022-03-13

**Authors:** Nga Yan Tse, Sicong Tu, Yu Chen, Jashelle Caga, Carol Dobson-Stone, John B. Kwok, Glenda M. Halliday, Rebekah M. Ahmed, John R. Hodges, Olivier Piguet, Matthew C. Kiernan, Emma M. Devenney

**Affiliations:** 1 The Brain and Mind Centre, University of Sydney; and Royal Prince Alfred Hospital, 94 Mallet Street, Camperdown, Sydney, NSW 2050 Australia; 2 School of Psychology and Brain and Mind Centre, The University of Sydney, Sydney, Australia; 3 Neuroscience Research Australia, Randwick, Australia; 4 Memory and Cognition Clinic, Department of Clinical Neurosciences, Royal Prince Alfred Hospital, Sydney, Australia

**Keywords:** Frontotemporal dementia, Amyotrophic lateral sclerosis, Motor neuron disease, Schizotypy, Neuropsychiatry

## Abstract

**Background:**

Psychiatric presentations similar to that observed in primary psychiatric disorders are well described across the amyotrophic lateral sclerosis–frontotemporal dementia (ALS–FTD) spectrum. Despite this, schizotypal personality traits associated with increased risks of clinical psychosis development and poor psychosocial outcomes have never been examined. The current study aimed to provide the first exploration of schizotypal traits and its neural underpinnings in the ALS–FTD spectrum to gain insights into a broader spectrum of psychiatric overlap with psychiatric disorders.

**Methods:**

Schizotypal traits were assessed using the targeted Schizotypal Personality Questionnaire in 99 participants (35 behavioural variant FTD, 10 ALS–FTD and 37 ALS patients, and 17 age-, sex- and education-matched healthy controls). Voxel-based morphometry analysis of whole-brain grey matter volume was conducted.

**Results:**

Relative to controls, pervasive schizotypal personality traits across positive and negative schizotypy and disorganised thought disorders were identified in behavioural variant FTD, ALS (with the exception of negative schizotypy) and ALS–FTDALS–FTD patients (all *p* < .013), suggesting the presence of a wide spectrum of subclinical schizotypal symptoms beyond classic psychotic symptoms. Atrophy in frontal, anterior cingulate and insular cortices, and caudate and thalamus was involved in positive schizotypy, while integrity of the cerebellum was associated with disorganised thought disorder traits.

**Conclusions:**

The frontal–striatal–limbic regions underpinning manifestation of schizotypy in the ALS–FTDALS–FTD spectrum are similar to that established in previous schizophrenia research. This finding expands the concept of a psychiatric overlap in ALS–FTD and schizophrenia, and suggests potentially common underlying mechanisms involving disruptions to frontal-striatal-limbic networks, warranting a transdiagnostic approach for future investigations.

**Supplementary Information:**

The online version contains supplementary material available at 10.1007/s00415-022-11049-3.

## Introduction

Frontotemporal dementia (FTD) and amyotrophic lateral sclerosis (ALS) can be considered as a continuum, with significant overlap across clinical, genetic and neuropathological features [1, 2]. Accumulating evidence suggests that psychosis can occur prior to the onset of typical cognitive, behavioural or motor symptoms in behavioural variant FTD (bvFTD) [3, 4] and ALS [5], as well as kindreds of ALS patients [6, 7]. Hallucinations and delusions, as classic positive symptoms of psychosis, have received the most research interest with a prevalence of up to 50% reported in patients with ALS–FTD and *C9orf72* associated ALS–FTD [8–10]. Since its discovery in 2011, there has been mounting evidence of a differential clinical presentation in FTD and ALS associated with the *C9orf72* repeat expansion characterised by a greater frequency of psychotic symptoms [11].

In addition to delusions and hallucinations, psychosis encompasses thought disorganisation and negative symptoms and that overlaps with the behavioural aspects of ALS–FTD. One study has examined the prevalence of all three subtypes of psychosis in bvFTD and found negative psychotic symptoms in 95.5% of bvFTD patients, followed by disorganised thought disorders (81.8%) and positive symptoms (22.7%)[12]. The prevalence of negative symptoms and disorganised thought have yet to be examined in ALS or ALS–FTD. While there is emerging evidence for more extensive overlap across ALS–FTD, *C9orf72* and neuropsychiatric disorders, including schizophrenia and autism spectrum disorder (ASD) in terms of shared genetic susceptibility, brain network disruption and co-morbidities [5, 13], the nature of overlap in psychotic symptoms remains poorly understood due to the lack of studies directly comparing these disorders. As such, the use of questionnaires validated in the psychiatric populations tapping into all subdomains of psychosis represents an important first step towards characterising the psychiatric overlap with ALS–FTD spectrum.

However, the use of traditional clinical scales to measure clinical or manifesting psychosis in ALS–FTDALS–FTD precludes the detection of subclinical psychotic symptoms that have been associated with increased risks of clinical psychosis development and have a detrimental impact on well-being and functioning [14–16]. In particular, schizotypal personality traits represent a constellation of subclinical psychosis symptoms (e.g. magical thinking, ideas of reference and odd beliefs) that generally mirror those seen in schizophrenia yet are insufficient to warrant a clinical diagnosis. Evidence suggests that these traits may confer a risk of developing clinical psychosis [14–16] and are associated with psychiatric co-morbidities such as persistence of suicidal ideation [17], mood and substance use disorders [16], as well as functional impairment [16]. Exploration of schizotypal traits in the ALS–FTD spectrum may expand our understanding of individual susceptibility to psychosis and the wider spectrum of overlapping symptomatology across neuropsychiatric and neurodegenerative conditions.

Studies exploring the neural substrates of psychosis in bvFTD and ALS remain surprisingly scant. The few existing neuroimaging studies are limited by the lack of subtyping of psychotic symptoms with a primary focus on classic hallucination and delusion symptoms. Atrophy in an extensive network of regions involving bilateral prefrontal and occipital cortices, right thalamus and left cerebellum has been associated with greater severity of hallucinations and delusions across patients with bvFTD and ALS–FTD [18].

Extrapolating from the extensive psychosis literature available together with evidence from the ALS–FTD psychosis literature, we hypothesised that subclinical psychosis traits could be associated with a distributed region of atrophy that centres on the fronto-striatal–limbic network. As such, the present study aims to present a detailed exploration of (i) a broad spectrum of schizotypal personality traits across the ALS–FTD spectrum using a comprehensive measure of positive schizotypy, negative schizotypy, and thought disorganisation as a measure of psychosis risk; and (ii) the association between grey matter volume reduction and the severity of schizotypal traits within each domain using voxel-based morphometry (VBM) analysis to establish the neural correlates of schizotypy subtypes.

## Materials and methods

### Participants

In total, 99 participants (35 bvFTD, 10 ALS–FTD and 37 ALS patients, and 17 healthy controls) were recruited from the FRONTIER Clinic and the FOREFRONT MND and FTD Clinic, the clinical research clinics specialising in younger-onset dementias and motor neurodegenerative syndromes, respectively; based at the Brain and Mind Centre, University of Sydney, Australia. Standardised diagnostic assessment comprised of: a medical and neurological examination, neuropsychological assessment, clinical interviews and blood sampling. All ALS patients received further neurophysiological examination including nerve conduction studies and transcranial magnetic stimulation. Functional impairment in ALS and ALS–FTD patients was measured using the revised ALS functional rating scale (ALSFRS-R [19]). ALS and ALS–FTD patients with both limb and bulbar onset were included in the study. Cognitive testing was conducted by trained neurologists or neuropsychologists and motor impairments were taken into account. For patients who were unable to verbalise, they were given the opportunity to type or write their responses. Patients who were unable to communicate by any method were not included in the study. None of the patients required non-invasive ventilation at the time of study completion. For timed measure in terms of the fluency subdomain on the ACE-III, the verbal fluency index that controls for motor speed was used [20].

Diagnosis was determined by multidisciplinary consensus by a senior neurologist, clinical neurophysiologist and clinical neuropsychologist according to the current clinical diagnostic criteria for bvFTD [21], ALS–FTD [22] and ALS [23, 24]. All healthy participants were matched for age, sex and education years, and scored above the cutoff for normal range (> 88/100) on the Addenbrooke’s Cognitive Examination (ACE-III) [25]. All controls also underwent blood sampling to ensure absence of common ALS–FTD-related genetic mutations including *C9orf72* repeat expansions, and mutation in the granulin (GRN) and microtubule-associated protein tau (*MAPT*) genes. Exclusion criteria for both patients and controls included the presence of other dementia syndrome, and/or pre-existing psychiatric disorders prior to disease onset.

### Standard protocol approvals, registrations, and patient consents

Ethical approval was obtained from the South Eastern Sydney Local Health District, the University of New South Wales ethics committees and the University of Sydney ethics committees. All the participants or their person responsible provided written, informed consent to participate in accordance with the Declaration of Helsinki.

### Blood sampling

All patients and controls underwent blood sampling to screen for a *C9ORF72* repeat expansion, and GRN and MAPT mutations. Genomic DNA was extracted from peripheral blood lymphocytes. Proband DNA samples were then screened for the hexanucleotide repeat expansions in the *C9ORF72* gene using a repeat primed polymerase chain reaction based on the protocol of Renton et al. [26] Samples were scored as positive if they harboured an allele with more than 30 repeats. Sanger sequencing was performed to identify mutations in *GRN* and *MAPT*.

### Assessment of psychotic features

Detailed psychiatric evaluation was conducted with the carer where the presence of psychosis was determined based on the definition of delusion and hallucinations outlined in the Fifth edition of The Diagnostic and Statistical Manual of Mental Disorders (DSM-5) [27]. All interviews were conducted by a cognitive and behavioural neurologist (E.D.) with experiences in psychiatric features in ALS–FTD.

To further characterise the pattern and nature of schizotypal traits, all patients completed the well-validated 74-item Schizotypal Personality Questionnaire (SPQ) [28, 29] in a yes/no format (with a score of 1 assigned to each yes response). The higher the score, the more severe the schizotypal traits in the domains of positive schizotypy (i.e. magical ideation, unusual perceptual experiences referential thinking and suspiciousness), negative schizotypy (i.e. social anxiety, lack of close friends, constricted affect, and suspiciousness), and disorganised thought disorder (i.e. odd speech and odd behaviour). All items on the SPQ can be found in supplementary Table 1.

### Statistical analysis

Data were analysed using SPSS Statistics, version 24.0 (IBM, Armonk, NY). The statistical significance level was set at *p* < 0.05 for all analyses unless otherwise specified. The assumption of normality was violated for most of the continuous variables based on Shapiro–Wilk results, therefore, non-parametric tests were used for all group comparisons unless otherwise specified. Differences in demographic (i.e., age and education) and SPQ subdomain scores (i.e. positive schizotypy, negative schizotypy and thought disorganisation) were examined between all groups using a Kruskal–Wallis test, followed by Mann–Whitney *U* post hoc comparisons with statistical significance set at a more conservative level of *p* < 0.013 for comparisons between disease groups (i.e., bvFTD, ALS–FTD, ALS and controls), and *p* < 0.017 for genetic status (i.e., *C9orf72* carriers, non-carriers and controls) and psychosis status (i.e. patients identified as with and without positive psychosis on clinical interview, and controls), following Bonferroni correction for multiple comparisons (i.e., 0.05 divided by the number of levels of independent variables). Categorical variable (i.e. sex) was examined using chi-squared tests. Difference in clinical variables specific to ALS and ALS–FTD (i.e. ALSFRS-R score and site of onset) were also examined using independent sample *t* test and Chi-square test, respectively.

To examine whether *C9orf72* results may be driven by particular diagnostic groups, the above analyses were repeated to compare demographic (i.e. sex, education years, age at scan), global cognitive (i.e., ACE total score), clinical (i.e., disease duration) and schizotypal (i.e. all three SPQ subdomain scores) characteristics between diagnostic subgroups with *C9orf72* repeat expansions.

To account for the potential effect of motor impairment on the display of negative schizotypal traits (with an emphasis on social withdrawal) and disorganised thought disorder (characterised by odd speech and odd behaviour) in ALS and ALS–FTD, Spearman’s correlation was conducted to examine the correlation between ALSFRS-R score and negative schizotypy and disorganisation subdomain scores, respectively.

This approach allows for exploration of the nature and pattern of schizotypal traits associated with different diagnostic and genetic cohorts, as well as examination of subclinical schizotypal features in those that were traditionally considered to have psychotic symptoms defined as the presence of either hallucination or delusion according to the Diagnostic and Statistical Manual of Mental Disorders, Fifth Edition (DSM-5) criteria [27].

## Imaging

### Brain imaging acquisition

A subset of participants (33 bvFTD, 7 ALS–FTD, 29 ALS, and 17 controls) underwent a whole-brain structural MRI with a 3 T General Electric (GE) scanner, fitted with a standard eight-channel head coil. T1-weighted sequences were acquired using the following protocol: matrix = 256 × 256, 200 slices, 1mm^2^ in-plane resolution, slice thickness = 1 mm, echo time = 2.6 ms, repetition time = 5.8 ms, flip angle = 8°.

### Imaging processing

All T1-weighted images were first visually inspected for the presence of any artefacts (e.g. head movements or poor contrast) by the investigators (N.Y.T and S.T.). As a result, ten ALS participants were excluded due to substantial head movement during image acquisition.

The final set of images (*n* = 76) were then analysed using VBM with Statistical Parametric Mapping version 12 (SPM12; Wellcome Department of Cognitive Neurology, London, UK) in Matlab R2018b (Mathworks, Natick, Massachusetts, USA) to examine whole-brain grey matter volumes. Firstly, T1-weighted images were segmented into five tissue probability maps in the native space. A Diffeomorphic Anatomical Registration using Exponentiated Lie (DARTEL) algebra template was then computed using the grey and white matter probability maps generated. Lastly, the grey matter probability maps were spatially normalised to the Montreal National Institute (MNI) space according to the transformation parameters from the DARTEL template. The resulting images were modulated and smoothed with a Gaussian kernel of 8 mm (full-width at half maximum).

### Imaging statistical analysis

Firstly, whole-brain grey matter intensity differences between each patient group and controls were investigated using a voxel-wise whole-brain general linear model (GLM) with total intracranial volume included as a nuisance variable. The total intracranial volume was assessed in native space prior to spatial normalisation by summing the thresholded grey matter, white matter and corticospinal fluid probability maps (threshold = 0.2) and counting non-zero voxels.

Next, correlations between whole-brain grey matter volumes and each of the SPQ subdomains were examined using GLM covariate analyses across all patient groups. Specifically, the SPQ positive schizotypy, negative schizotypy, and thought disorganisation subdomain scores were entered as covariates in three separate GLMs with total intracranial volume as a nuisance variable to account for the effect of different head sizes. All analyses were corrected for cluster-extent multiple comparisons at *p* < 0.05, with a cluster-forming threshold of *p* < 0.001. All clusters were anatomically defined and visualised using xjView toolbox (http://www.alivelearn.net/xjview).

## Results

### Demographic characteristics

Comparisons between patient groups revealed significantly longer disease duration in bvFTD compared to both ALS (*p* < 0.001) and ALS–FTD (*p* = 0.002) groups, while ALS and ALS–FTD groups were not found to differ in disease duration, ALSFRS-R score or site of onset (*p* > 0.05; Table 1). No significant group differences were observed in sex, education or age across all groups (all *p* values > 0.05; Table 1). Both bvFTD and ALS–FTD groups demonstrated significantly lower ACE-III total scores compared to controls (both *p* < 0.001) and ALS patients (*p* < 0.001 and *p* = 0.003).

**Table 1:** Demographic characteristics across diagnosis groups

	Controls (*n* = 17)	bvFTD (*n* = 35)	ALS–FTD (*n* = 10)	ALS (*n* = 37)	*H*	*p*	Post-hoc
Sex (M/F)	8/9	26/9	8/2	28/9	5.664^a^	.129	–
Education (years)	13.8 ± 2.3	12.8 ± 3.3	12.5 ± 2.2	13.3 ± 3.1	2.798	.424	–
Age (years)	62.0 ± 9.7	61.9 ± 7.1	58.4 ± 11.2	62.8 ± 8.8	1.25	.741	–
ACE total (/100)	95.8 ± 2.4	79.9 ± 12.3	84.3 ± 8.2	92.3 ± 5.4	42.795	< .001	Control > bvFTD, ALS–FTD
							ALS > bvFTD, ALS–FTD
Disease duration (months)	–	75.6 ± 50.0	29.6 ± 18.6	30.7 ± 31.4	28.998	< .001	bvFTD > ALS, ALS–FTD
ALSFRS-R	–	–	39.6 ± 6.8	39.8 ± 4.7	.123	.903	–
Onset site (limb/bulbar)	–	–	6/4	24/13	.081^a^	.776	–

Means ± standard deviation. ^a^Chi-square value. *H* = Kruskal–Wallis test statistic; post hoc = Mann–Whitney *U* post hoc comparison results

*ACE*  Addenbrooke’s Cognitive Examination, *ALSFRS-R*  the revised ALS functional rating scale, *bvFTD* behavioral variant frontotemporal dementia, *ALS–FTD*  amyotrophic lateral sclerosis–frontotemporal dementia, *ALS* amyotrophic lateral sclerosis

Comparisons between *C9orf72* expansion carriers (*n* = 14), noncarriers (*n* = 68) and controls (*n* = 17) revealed no significant differences in age or education years (supplementary Table 2). Sex distribution, however, differed in the control group with a greater distribution of female participants (*p* = 0.016). Compared with controls, both *C9orf72* carrier and noncarrier groups demonstrated significantly lower ACE-III total scores (both *p* < 0.001), while both patient groups demonstrated comparable ACE-III total scores (*p* = 0.151).

No significant differences were found in demographic or clinical variables (i.e. age, sex, education and disease duration; supplementary Table 3) across patients with and without psychosis, and controls. Patients with and without psychosis performed significantly worse on the ACE-III compared to controls (*p* < 0.001).

## Psychotic features

### *ALS-FTD* spectrum

Schizotypal features were evident across the ALS–FTD spectrum (Table 2). Relative to controls, both bvFTD and ALS–FTD groups demonstrated significantly higher positive schizotypy (*p* = 0.007; *p* < 0.001), negative schizotypy (*p* = 0.001; *p* < 0.001) and disorganisation (*p* = 0.004; *p* < 0.001) subdomain scores. Similarly, significantly higher positive schizotypy (*p* = 0.012) and thought disorganisation (*p* = 0.01) subdomain scores were observed in the ALS group. Significantly higher positive (*p* = 0.001) and negative (*p* = 0.010) schizotypy and disorganisation (*p* = 0.001) scores were further revealed in the ALS–FTD group compared to the ALS group.

**Table 2:** Schizotypal characteristics between controls, and diagnosis groups, *C9orf72* expansion status, and the presence of psychotic features

	Controls (*n* = 17)	bvFTD (*n* = 35)	ALS–FTD (*n* = 10)	ALS (*n* = 37)	C9 carriers (*n* = 14)	Non-carriers (*n* = 68)	Patients with psychosis (*n* = 25)	Patients without psychosis (*n* = 57)	*p*^*a*^	Post-hoc
*SPQ*										
Positive schizotypy	1.2 (1.3)	6.3 (7.1)	9.8 (7.2)	3.2 (2.7)	8.9 (5.6)	4.6 (5.8)	10.4 (6.3)	3.1 (4.1)	< .001	bvFTD, ALS–FTD, ALS > Control
									< .001	ALS–FTD > ALS
									< .001	C9, Noncarriers > Control
									< .001	C9 > Noncarriers
									< .001	With psychosis > Without psychosis, Control
Negative schizotypy	2.8 (2.3)	9.7 (8.5)	12.4 (8.8)	5.8 (4.9)	12.1 (8.2)	7.5 (7.1)	12.6 (8.6)	6.4 (6.0)	< .001	bvFTD, ALS–FTD > Control
									< .001	C9, Noncarriers > Control
										ALS–FTD > ALS
									< .001	With psychosis > Without psychosis, Control
									< .001	Without psychosis > Control
Disorganised thought disorder	1.1 (1.7)	4.0 (4.1)	6.7 (4.4)	2.4 (2.2)	4.4 (2.5)	3.4 (3.8)	5.7 (4.1)	2.7 (3.0)	< .001	bvFTD, ALS–FTD, ALS > Control
										ALS–FTD > ALS
									.001	C9, Noncarriers > Control
									< .001	With psychosis > Without psychosis, Control

Means (standard deviation). ^a^*p* value for Kruskal–Wallis test statistics. Post hoc = Mann–Whitney *U* post hoc comparison results

*bvFTD*  behavioral variant frontotemporal dementia, *ALS–FTD* amyotrophic lateral sclerosis–frontotemporal dementia, *ALS*  amyotrophic lateral sclerosis, *SPQ*  Schizotypal Personality Questionnaire

In terms of the potential contribution of motor impairment to negative schizotypy and disorganisation expression, exploratory analyses did not reveal significant correlations between ALSFRS-R score and negative schizotypy or disorganisation subdomain scores (all *p* > 0.05; supplementary Table 4).

### *C9orf72* expansion carriers compared to noncarriers

Compared with controls, both *C9orf72* expansion carriers and noncarriers demonstrated significantly higher SPQ scores across the positive schizotypy (*p* < 0.001; *p* = 0.008), negative schizotypy (*p* < 0.001; *p* = 0.003) and disorganisation (*p* < 0.001; *p* = 0.004) subdomains (Table 2). Disproportionately higher positive schizotypy scores were further revealed in *C9orf72* carriers compared to noncarriers (*p* = 0.003).

Additional analyses did not reveal significant differences in demographic (i.e. sex, education years, age at scan; supplementary Table 5), global cognitive (i.e., ACE total score), clinical (i.e., disease duration) or schizotypal (i.e., all three SPQ subdomain scores) characteristics between diagnostic subgroups with *C9orf72* repeat expansion, suggesting that the *C9orf72* subanalysis results are unlikely to be driven by any of the diagnostic groups and representative of the ALS–FTD spectrum.

### Patients presenting with positive psychosis compared to those without psychosis

Patients presenting with positive psychosis symptoms as determined by a traditional carer-based clinical interview displayed significantly higher scores across the positive schizotypy, negative schizotypy and disorganisation subdomains compared to those without positive psychosis and controls (all *p* values ≤ 0.001; Table 2). Furthermore, significantly higher negative schizotypy scores were revealed in patients without psychosis when compared to controls (*p* = 0.013).

### VBM results

Widespread cortical grey matter intensity reduction that are largely consistent with previous reports in the literature was found in bvFTD (supplementary Fig. 1), ALS–FTD (supplementary Fig. 2) and ALS (supplementary Fig. 3) when compared to controls separately [30].

**Fig. 1:**
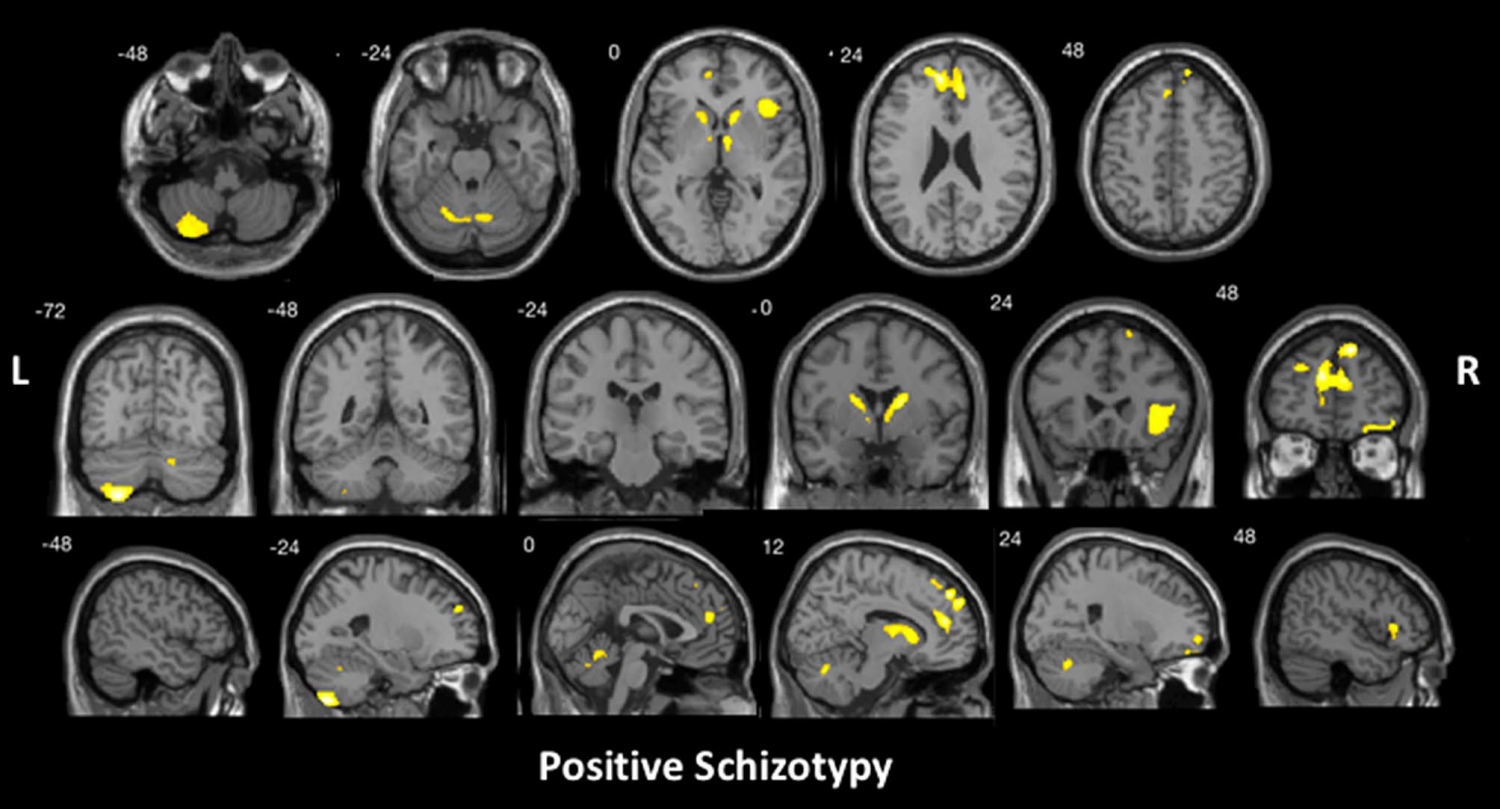
Brain regions that were significantly correlated with SPQ positive schizotypy subdomain scores for the ALS–FTD spectrum, controlling for total intracranial volume, using voxel-based morphometry analyses. Clusters were corrected for cluster-extent multiple comparisons at *p* < .05, with a cluster-forming threshold of *p* < .001. *L*  left, *R*  right

**Fig. 2:**
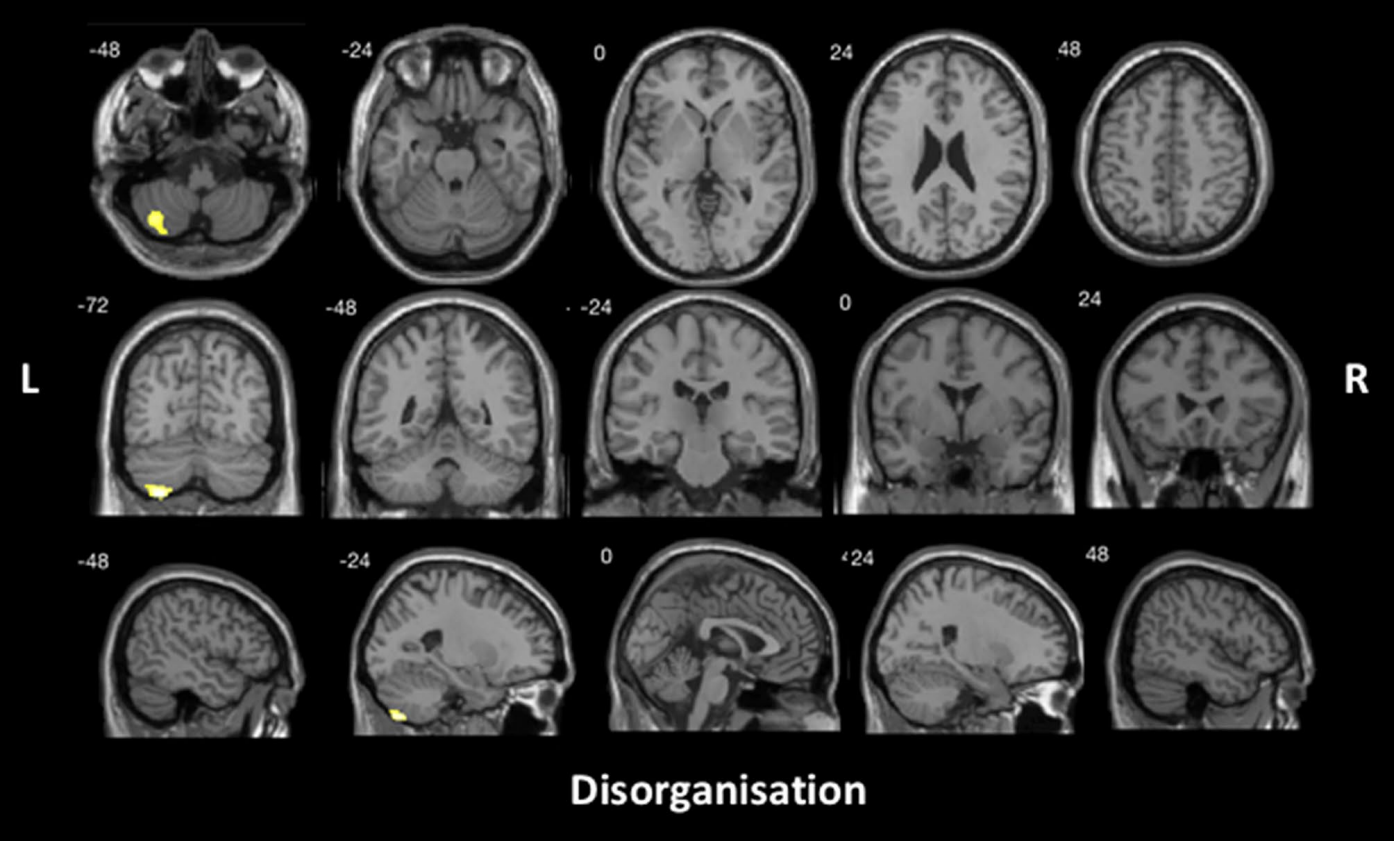
Brain regions that were significantly correlated with SPQ disorganised thought disorder subdomain scores across the ALS–FTD spectrum, controlling for total intracranial volume, using voxel-based morphometry analyses. Clusters were corrected for cluster-extent multiple comparisons at *p* < .05, with a cluster-forming threshold of *p* < .001. *L*  left, *R*  right

With all patient groups combined, positive schizotypy subdomain scores were found to be significantly associated with atrophy in bilateral superior and medial frontal gyrus, posterior cerebellum, and right anterior cingulate, insular cortex, inferior frontal gyrus, frontal orbital cortex, caudate and thalamus (Fig. 1 and Table 3). Significant correlations emerged between the cerebellum and disorganisation subdomain scores (Fig. 2 and Table 3). No significant correlation was identified within the negative schizotypy subdomain.

**Table 3:** Voxel-based morphometry results of significant grey matter intensity reduction that correlates significantly with SPQ subdomain scores

SPQ subdomains	Cluster size, voxels	MNI coordinates			*T* value	H	Regions
		X	Y	Z			
Positive schizotypy	3321	15	48	44	5.62	Right	Superior frontal gyrus extending into medial frontal gyrus and anterior cingulate, and left medial and superior frontal gyrus
	1365	– 26	– 72	– 56	5.53	Left	Cerebellum lobules VIII, VIIb and Crus II
	1429	18	– 2	15	4.82	Right	Caudate into thalamus
	1175	40	26	2	4.32	Right	Insular cortex into inferior frontal gyrus
	744	15	– 69	– 27	3.85	Right	Cerebellum lobule VI into left lobule VI and vermis of lobules IV and V
	669	34	60	– 14	4.61	Right	Frontal orbital cortex into superior and middle frontal gyrus
	673	– 14	14	3	4.05	Left	Caudate
Disorganised thought disorder	771	– 27	– 75	– 54	4.38	Left	Cerebellum lobule VIIb into lobules VIII and Crus II

Results reported at cluster-level FWE-corrected *p* < .05 (cluster-forming threshold, *p* < .001)

Total intracranial volume was included as a nuisance variable in all analyses

*H* hemisphere, *MNI*  Montreal Neurological Institute, *SPQ*  the Schizotypal Personality Questionnaire

## Discussion

Using a targeted measure of schizotypal traits and in combination with VBM analyses, we present the first comprehensive examination of a broad spectrum of subclinical psychosis symptoms in the ALS–FTD continuum, providing novel insights into the nature and pattern of schizotypy including the influence of genetic abnormalities on the manifestation of these symptoms with potential clinical and functional implications.

Capturing the full spectrum of schizotypal traits, higher expression of positive schizotypy relative to healthy controls were revealed in not only bvFTD and ALS–FTD, but also ALS patients, shedding light on the presence of positive schizotypal features in ALS, traditionally characterised by less severe behavioural disturbance. A higher frequency of positive schizotypy symptoms was found in *C9orf72* compared to noncarriers, in line with previous findings of prominent neuropsychiatric presentation across bvFTD [11, 31, 32] and ALS [33] with *C9orf72.* These findings may have important clinical implications given the associated risk of progression into psychosis. In particular, positive schizotypal personality traits have been linked to the early prodromal stage of schizophrenia [34], representing a risk factor for schizophrenia [35, 36]. Therefore, clinical interview in conjunction with a targeted questionnaire (e.g. the SPQ) probing subtle psychotic-like features may offer avenues for detection of vulnerability to onset of frank psychosis.

Negative schizotypy was present in patients without frank positive psychosis, indicating that other subdomains of schizotypal features can present even in those without delusions or hallucinations. This is perhaps unsurprising given the overlap between the negative schizotypy and disorganisation symptoms and the behavioural (e.g. apathy) [21, 22] and motor features associated with the ALS–FTD spectrum, suggesting that psychiatric features overlap with behavioural features at the clinical level, likely as consequences of the disease process. Of further interest is the significant overlap in negative schizotypy symptoms implicated on the SPQ and hallmark features of ASD characterised by social withdrawal and difficulties with verbal and nonverbal communication in social interactions. The current findings consolidate the growing evidence of shared symptomatology between FTD and other neuropsychiatric conditions such as schizophrenia and ASD in addition to genetic susceptibility, brain network disruption and co-morbidities [5, 13].

Our neuroimaging analyses revealed that the breakdown of the fronto-striatal–limbic system plays a central role in the manifestation of positive schizotypy. Largely commensurate with the FTD literature [18], we identified a similar and wider network of regions including bilateral frontal, cerebellar regions, in addition to limbic and striatal structures including right anterior cingulate and insular cortices, caudate and thalamus in the manifestation of positive schizotypal traits. Fronto-striatal-limbic abnormalities have been extensively reported in schizophrenia research [37–39]. Abnormal striatal activation and disruption of fronto-striatal connectivity have previously been identified in individuals presenting with schizotypal personality traits [40], and unaffected first-degree relatives [41], respectively, supporting occurrence of neuropathological changes during the prodromal stage. Similar disruption of the fronto-striatal–limbic network in terms of grey matter volume loss in bilateral fronto-limbic regions (including anterior cingulate and insula) have been commonly found in both bvFTD and ALS [42], with more widespread frontal and limbic (including the thalamus) atrophy and additional striatal (including the caudate and putamen) atrophy further identified in bvFTD [43, 44]. Commonalities in the fronto-striatal–limbic abnormalities identified between ALS–FTD and schizophrenia suggests that the emergence of psychotic symptoms may be attributable to a similar underlying pathological mechanism. In other words, the regions implicated in schizophrenia may be applicable to the wider ALS–FTD population contributing to the development of shared psychotic experiences.

The posterior cerebellum was implicated in disorganised thought disorders. Cerebellum had traditionally been regarded as solely responsible for motor functions in coordination and execution of movement [45]. However, there has been robust evidence of cerebellar contribution to cognitive impairments in various clinical populations including all variants of FTD [46], ALS–FTD [47] and other syndromes of frontotemporal lobar degeneration [48]. The resulting cognitive and emotional disturbance are attributable to disruption of the connectivity between cerebellum and cortical areas subserving higher-order cognitive and emotion regulation functions [49, 50], leading to dysmetria of thought (i.e., discoordination of cognitive and emotional processing) analogous to discoordination of movements observed in cerebellar motor syndromes [51]. This suggests that cerebellar atrophy likely contributes to the emergence of higher-order thought disorganisation in ALS–FTD similar to that observed in schizophrenia, over and beyond its impact on motor functions.

While the current study provides the first detailed exploration of schizotypal traits across the ALS–FTD spectrum, several issues need to be considered. Firstly, the use of a self-report measure of schizotypal traits raises potential concern regarding the accuracy of self-evaluations. Given that current study is at the forefront of research on the potential overlap across psychiatric and neuropsychiatric conditions, our choice of measure is limited by the unavailability of an informant-based schizotypy measure that translates across psychiatric and neurodegenerative conditions. This is perhaps unsurprising given the challenges associated with detecting subtle psychosis-like internal states from a third-person perspective. The significant differences across all subdomains of schizotypy between patients classified as presenting with psychosis compared to those without through clinical interview conducted in the presence of a family member, however, supports the association between SPQ scores and clinically ascertained psychotic symptoms and provides supporting evidence of the sensitivity and validity of the SPQ. The use of a self-report measure is further supported by recent observations of a high level of correlation between patient- and carer-report versions of a psychosis measure in the ALS–FTD spectrum [52]. Nonetheless, the current findings remain an important first step towards establishing the utility of examining schizotypal symptoms in the ALS–FTD spectrum. Future efforts into developing a measure of schizotypal traits and psychotic features tailored to the FTD and/or ALS populations is warranted to facilitate its wider use in the clinical setting.

Further, the absence of significant association between brain structural changes and negative schizotypy may be related to the heavy focus on social factors on the SPQ negative psychosis subdomain where the majority of the questions concern social functioning (i.e. social communication difficulties, social anxiety and paranoid ideation) without tapping into classic negative psychotic symptoms such as loss of motivation and/or reduced initiation. This would appear to be in line with previous findings of a lack of interpersonal sensitivity and paranoid ideation in 111 ALS patients despite the presence of extensive psychological symptoms, including somatisation, anxiety, phobic anxiety, and depression [53]. In light of this, future studies will benefit from the additional use of a more comprehensive psychological measure that targets a wider dimension of psychological characteristics beyond the context of social functioning to comprehensively chart the neuropsychiatric profile across the ALS–FTD spectrum. Study of schizotypal features in pre-symptomatic carriers may provide insight into the time point that these features emerge and also determine whether they are a pre-requisite to frank psychosis in this population. Similarly, longitudinal assessment of structural brain changes beginning prior to onset of psychotic symptoms will help delineate the role of particular brain regions in the development of schizotypal traits, and subsequently, clinical psychosis at different disease stages. Lastly, future studies may consider the use of functional neuroimaging to examine functional connectivity in light of the increasing recognition that the manifestation of psychiatric disorders are generated from network disruption [54]. Moreover, this will also offer direct insight into whether different subtypes of schizotypal traits are underpinned by distinct functional networks.

In a similar vein, the absence of significant differences in negative schizotypy in the ALS group may be attributable to the relative preserved cognitive functioning in the current cohort of ALS patients given the recent finding of poorer cognitive performance in those with a personal or family history of neuropsychiatric disorders. It will therefore be beneficial for future studies with a larger sample of ALS patients to subclassify patients on the basis of presence of behavioural and/or cognitive impairment in accordance with the Strong et al. criteria [22] to assist in disentangling the relationship between the prevalence of subtypes of schizotypal symptoms and behavioural and cognitive deficits.

## Conclusion

The nature of schizotypal traits in the ALS–FTD continuum is not limited to classic hallucinations or delusions with negative schizotypy and/or disorganised thought disorders evident across bvFTD, ALS–FTD and ALS. Current neuroimaging findings confirmed the involvement of fronto-striatal–limbic atrophy and cerebellar atrophy in the manifestation of positive psychosis and disorganised thoughts, respectively. This is in line with the frontal-striatal-limbic involvement reported extensively in previous schizophrenia research, suggesting that the shared psychotic features between the ALS–FTD spectrum and schizophrenia may be underpinned by disruption of a common network of brain regions. In addition to providing further insight into disease pathophysiology and better definition of clinical phenotype [55, 56], the present study raises further consideration of directed therapeutic interventions across these patient cohorts[57].

## Supplementary Information

Below is the link to the electronic supplementary material.

Supplementary file1 (DOCX 1091 KB)

## Data Availability

The data that support the findings of this study are available from the corresponding author on reasonable request.
