# Supplementary material for: Schizotypal traits across the amyotrophic lateral sclerosis–frontotemporal dementia spectrum: pathomechanistic insights
Source: J Neurol. 2022 Mar 13;269(8):4241–52. doi: 10.1007/s00415-022-11049-3 (PMC9294025; doi:10.1007/s00415-022-11049-3)
Supplement: Supplementary file 1 — Supplementary file1 (DOCX 1091 KB) [file 415_2022_11049_MOESM1_ESM.docx]

**Supplementary** **Table 1. Items of each SPQ subdomain:**

| **Positive schizotypy** |  | |
| --- | --- | --- |
| ***Magical ideation***  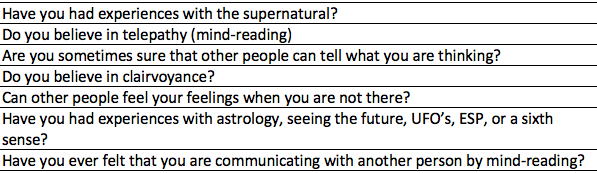 | |  |
| ***Unusual perceptual experiences***  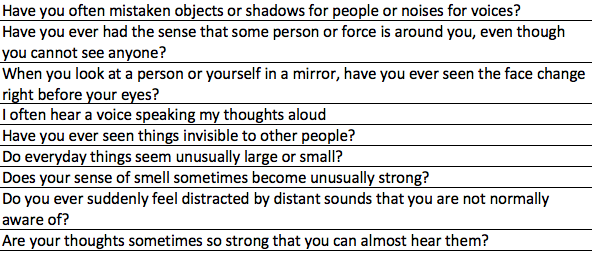 | |  |
| ***Referential thinking***  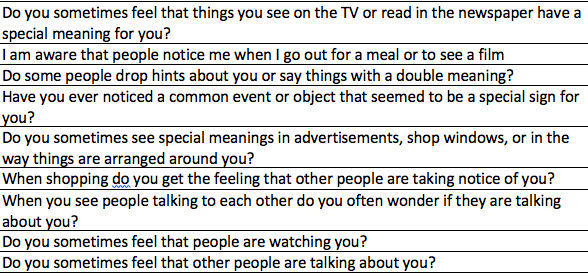 | |  |
| ***Suspiciousness***  *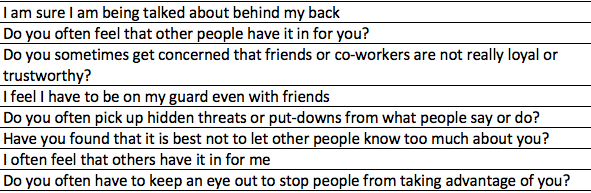* |  | |
| **Negative schizotypy** |  | |
| ***Social anxiety***  *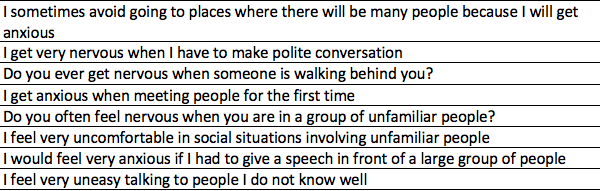* | |  |
| ***Lack of close friends***  *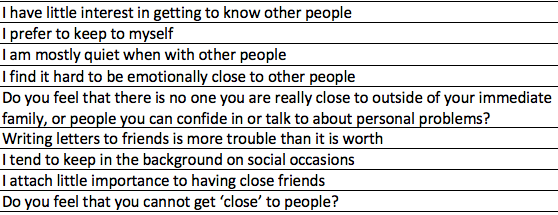* | |  |
| ***Constricted affect***  *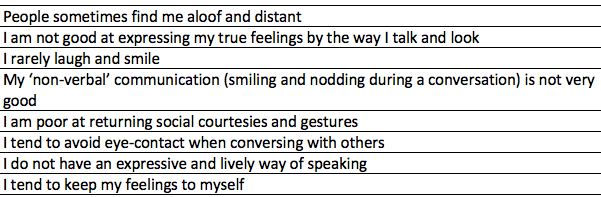* | |  |
| ***Suspiciousness***  *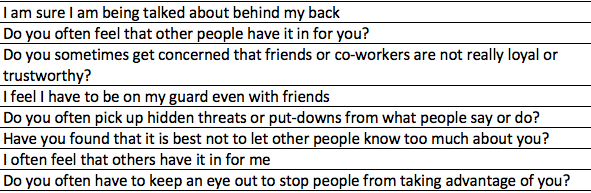* | |  |
| **Disorganisation** |  | |
| ***Odd speech***  *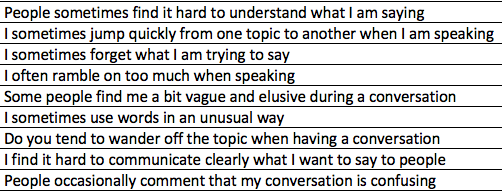* | |  |
| ***Odd behaviour***  *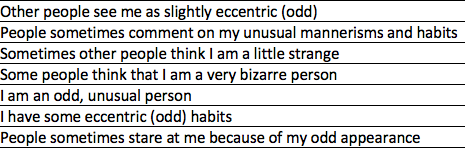* | |  |

SPQ = Schizotypal Personality Questionnaire.


**Supplementary** **Table 2.** Demographic characteristics between patients with and without *C9* expansion and controls

|  | **C9 carriers**  **(*n* = 14)** | **Noncarriers**  **(*n* = 68)** | **Controls**  **(*n* = 17)** | ***H*** | ***p*** | **Post-hoc** |
| --- | --- | --- | --- | --- | --- | --- |
| Sex (M/F) | 9/5 | 53/15 | 8/9 | 6.587^a^ | .037^a^ | Control |
| Education (years) | 12.9±2.5 | 13.0±3.2 | 13.8±2.3 | 2.099 | .350 | - |
| Age (years) | 60.5±7.9 | 62.2±8.6 | 62.0±9.7 | .591 | .744 | - |
| ACE Total (/100) | 81.1±14.7 | 87.1±9.8 | 95.8±2.4 | 20.084 | <.001 | Control>Patients |
| Disease duration (months) | 53.6±49.2 | 50.0±45.3 | - | 340.5^b^ | .635^b^ | - |

Means ± standard deviation. ^a^Chi-square value. ^b^Mann-Whitney *U* test.

*H* = Kruskal-Wallis test statistic; Post-hoc = Mann-Whitney *U* post-hoc comparison results.

ACE = Addenbrooke’s Cognitive Examination.

**Supplementary** **Table 3.** Demographic characteristics between patients with and without psychotic features and controls

|  | **Patients with psychosis**  **(*n* = 25)** | **Patients without psychosis**  **(*n* = 57)** | **Controls**  **(*n* = 17)** | ***H*** | ***p*** | **Post-hoc** |
| --- | --- | --- | --- | --- | --- | --- |
| Sex (M/F) | 19/6 | 43/14 | 8/9 | 5.544^a^ | .063 | - |
| Education (years) | 13.3±3.1 | 12.8±3.1 | 13.8±2.3 | 2.42 | .298 | - |
| Age (years) | 60.1±8.8 | 62.7±8.2 | 62.0±9.7 | 1.159 | .56 | - |
| ACE Total (/100) | 81.4±14.3 | 88.1±8.3 | 95.8±2.4 | 21.63 | <.001 | Control>Patients |
| Disease duration (months) | 48.7±41.0 | 51.2±47.5 | - | 611.5^b^ | .865 | - |

Means ± standard deviation. ^a^Chi-square value. ^b^Mann-Whitney *U* test. *H* = Kruskal-Wallis test statistic; Post-hoc = Mann-Whitney *U* post-hoc comparison results.

ACE = Addenbrooke’s Cognitive Examination.

**Supplementary Table 4.** Spearman’s Correlations between ALSFRS-R and SPQ negative schizotypy and disorganisation subdomains

|  | **ALSFRS-R** | ***p*** |
| --- | --- | --- |
| **SPQ** |  |  |
| Negative schizotypy | -.144 | .380 |
| Disorganisation | -.238 | .144 |

ALSFRS-R = the revised ALS functional rating scale; SPQ = Schizotypal Personality Questionnaire.

**Supplementary Table 5.** Demographic, clinical, and schizotypal characteristics across diagnosis groups with *C9orf72* expansions

|  | **bvFTD**  **(*n* = 7)** | **ALS-FTD**  **(*n* = 5)** | **ALS**  **(*n* = 2)** | ***H*** | ***p*** |
| --- | --- | --- | --- | --- | --- |
| Sex (M/F) | 4/3 | 2/0 | 3/2 | 1.307a | .520 |
| Education (years) | 12.57±3.10 | 12.75±1.71 | 14.50±2.12 | 1.505 | .471 |
| Age (years) | 60.43±7.53 | 61.20±9.96 | 59.00±8.49 | .329 | .848 |
| ACE Total (/100) | 76.86±17.98 | 81.40±9.07 | 95.50±2.12 | 3.341 | .188 |
| Disease duration (months) | 67.71±56.59 | 32.00±23.64 | 20.00±0.00 | 3.264 | .196 |
| SPQ |  |  |  |  |  |
| Positive schizotypy | 9.29±6.82 | 10.00±4.30 | 4.50±2.12 | 2.046 | .360 |
| Negative schizotypy | 12.14±10.68 | 9.20±3.42 | 19.50±0.71 | 2.184 | .336 |
| Disorganised  thought disorder | 4.29±2.81 | 4.60±2.70 | 4.00±1.41 | .457 | .796 |

Means ± standard deviation. ^a^Chi-square value. *H* = Kruskal-Wallis test statistic; Post-hoc = Mann-Whitney *U* post-hoc comparison results.

ACE = Addenbrooke’s Cognitive Examination; bvFTD = behavioral variant Frontotemporal Dementia; ALS-FTD = Amyotrophic Lateral Sclerosis-Frontotemporal Dementia; ALS = Amyotrophic Lateral Sclerosis; SPQ = Schizotypal Personality Questionnaire.


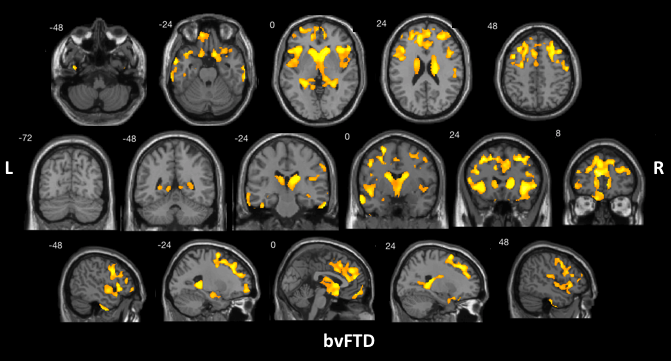
 **Supplementary** **Fig. 1** Voxel-based morphometry analyses with total intracranial volume included as a nuisance variable showing whole brain atrophy in contrasts between bvFTD and controls. Shaded voxels show regions that were significant in the analyses at the threshold of clusters corrected for cluster-extent multiple comparisons at *p* < .05, with a cluster-forming threshold of *p* < .001. bvFTD = behavioural variant frontotemporal dementia; L = Left; R = Right.


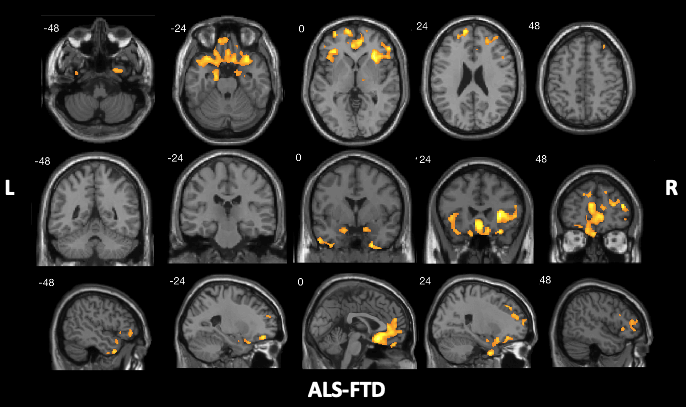


**Supplementary** **Fig. 2** Voxel-based morphometry analyses with total intracranial volume included as a nuisance variable showing whole brain atrophy in contrasts between ALS-FTD and controls. Shaded voxels show regions that were significant in the analyses at the threshold of clusters corrected for cluster-extent multiple comparisons at *p* < .05, with a cluster-forming threshold of *p* < .001. ALS-FTD = Amyotrophic Lateral Sclerosis-Frontotemporal Dementia; L = Left; R = Right.


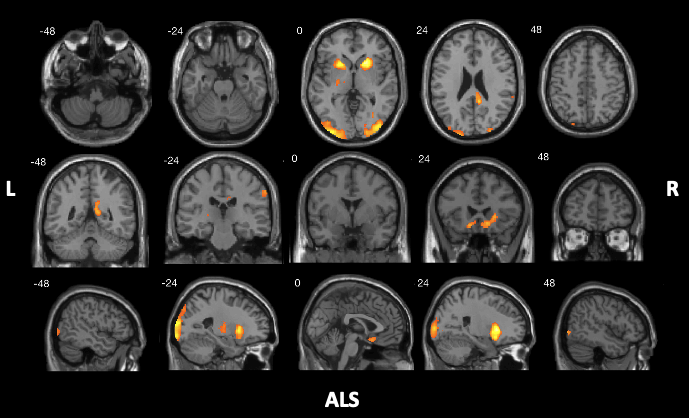


**Supplementary** **Fig. 3** Voxel-based morphometry analyses with total intracranial volume included as a nuisance variable showing whole brain atrophy in contrasts between ALS and controls. Shaded voxels show regions that were significant in the analyses at the threshold of clusters corrected for cluster-extent multiple comparisons at *p* < .05, with a cluster-forming threshold of *p* < .001. ALS = Amyotrophic Lateral Sclerosis; L = Left; R = Right.
